# Parental beliefs and practice of spiritual methods for their sick children at a tertiary care hospital of Pakistan- a cross sectional questionnaire study

**DOI:** 10.1186/s12906-016-0986-3

**Published:** 2016-01-13

**Authors:** Ghulam Mustafa, Nadir Bashir, Muhammad Aslam

**Affiliations:** 1 Department of Pediatrics, Nishtar Medical College, Multan, Pakistan; 2 Department of Pediatrics, Nishtar Hospital, Multan, Pakistan; 3 Department of Statistics, Bahauddin Zakariya University, Multan, Pakistan

**Keywords:** Complementary and alternative medicine, Knowledge, Attitude and practice, Spiritual, Taveez, Beliefs, Children

## Abstract

**Background:**

Complementary and alternative medicine (CAM) comprises a variety of health care systems, practices, and products that are not usually thought to be part of allopathic medicine. This study investigated the parental beliefs and practices for use of spiritual methods in the treatment and early recovery of their children.

**Methods:**

We performed a cross-sectional, descriptive study with convenience sampling of parents/caregivers of sick children who were admitted to the Children’s Hospital in Multan. A trained interviewer collected the data.

**Results:**

A total of 1280 forms were analyzed. The majority of respondents were mothers (1053, 82.4 %), they resided in Multan (817, 63.8 %), and were not educated (754, 58.9 %). A total of 420 (32.8 %) respondents had a low socioeconomic background, 601 (47 %) were middle class, and 259 (20.2 %) were upper class. Grandmothers/mothers advised spiritual methods in the majority of respondents (605, 85.9 %). The parents used a variety of spiritual methods in 704 (55 %) children. Economic status and education showed an inverse relation with the use of CAM. A total of 809 (63.2 %) respondents believed that only a drug would heal the disease, while 575 (44.9 %) believed that spiritual methods have a 25 %–50 % role in healing. A total of 1269 (99.1 %) respondents believed that allopathic drugs are needed for healing, while only 0.9 % considered otherwise.

**Conclusion:**

The majority of people believe that CAM is a contributory factor towards healing and does not interfere with allopathic treatment.

**Electronic supplementary material:**

The online version of this article (doi:10.1186/s12906-016-0986-3) contains supplementary material, which is available to authorized users.

## Background

The National Center for Complementary and Alternative Medicine of the National Institutes of Health defines complementary and alternative medicine (CAM) as “a group of diverse medical and health care systems, practices, and products that are not presently considered to be part of conventional Western medicine” [1]. CAM represents care that is centered on the patients, and it comprises spiritual, biological, social, environmental, and psychological health. CAM includes conventional and complementary treatments that have scientific proof of safety and efficacy. CAM fosters health for a person in the context of his/her family and society [1]. Therefore, CAM involves a collection of modalities practiced by communities.

The spiritual approaches of healing are designated as a part of CAM [2]. The American Academy of Pediatrics has included categorization of CAM as promoted by the National Center for Complementary and Alternative Medicine, and clusters prayers/spiritual treatment under the heading of mind–body medicine [3]. Spiritual treatment is used among adults [4], and according to a few reports, is a widespread complementary therapy in the United States [5]. A previous study showed that 82 % people have faith in healing outcomes of spirituality [6] and nearly 88.7 % practice it for various problems [7]. This situation is similar when applied to the parents of children with various illnesses. Almost two thirds [8] of parents use some form of spiritual remedy that they consider appropriate for their children during their illness [9].

Spiritual/religious practices can contribute to decreased stress, an increased sense of wellbeing, and improved functioning of the immune system [3]. Many families consider spiritual healing as a practice that is complementary to medical care rather than a substitution for it [6]. Although CAM is common in Pakistan [10–15], there is a lack of literature on spiritual methods of CAM. Parents engage these methods routinely as a preventive tool for the health of their children and to save them from the “evil effects” of the environment [10]. How parents of children with various illnesses draw upon their spiritual beliefs and practices when coping with their children’s illnesses needs to be investigated [16].

Therefore, this study aimed to assess parental beliefs on spiritual methods, their beliefs about the relation of allopathic drugs with spiritual methods, and the practice of spiritual methods before or during the illness of their children.

## Methods

### Study design and site

This was a descriptive, cross-sectional study. We conducted a knowledge, attitude, and practices (KAP) survey at the Children’s Hospital and the Institute of Child Health, Multan. This is a tertiary care center with 150 in-patient capacity. Because this hospital is the only sub-specialty hospital in the region, it serves the majority of the pediatric population of the south Punjab. As a public sector hospital, patients from all socioeconomic strata attend the hospital.

### Sample size and data collection

The proportion of CAM use by the general population of Pakistan is 51.7 % [17]. Therefore, with a 95 % power of the test and a 95 % confidence interval, the computed sample size was 1280. This sample size was calculated assuming a 5 % sample error, 50 % variance, and adjusting for a 50 % non-response rate.‬‬‬‬‬‬‬‬‬‬

The target population in this study was caregivers of children who were admitted in various wards of the Children’s Hospital and the Institute of Child Health, Multan. The caregivers of children, who consented for interviews, were eligible to participate in the study. The convenience sampling technique was used to select caregivers for the interviews. Information was collected using face-to-face interviews with a structured, pre-tested questionnaire. Informed consent for participation in the survey was obtained from the parents/caregivers of the children. A research assistant was fully trained for terminologies used in the questionnaire. He asked the caregivers questions and then filled out the questionnaire. After filling out the whole form, he repeated the questions and answers aloud to the caregivers to confirm the correctness of the answers. Confidentiality was strictly maintained throughout the process of data collection, entry, and analysis. The data collection time period was 8 months (September 2007 to March 2008).

### Questionnaire

#### Development

Currently, there is no standard survey instrument for assessing pediatric CAM use. Therefore, a questionnaire was developed to allow for the spiritual aspects of KAP, according to the established methodology. Ten questions were developed by the investigative team to address gaps in the knowledge of spiritual methods used in children. The draft questions were based on the prior experience of investigators, and input from colleagues, peers, and patients. The initial questionnaire was expanded by incorporation of new aspects that were encountered during an extensive literature search. The prepared draft was subjected to pilot testing to establish concept validity in a convenience sample of 30 respondents who were staff and parents of the Children’s Hospital. Experts in CAM and pediatrics reviewed the results and no changes were deemed necessary in the questionnaire. The results of the pilot testing were not included in the final data analysis. The language of the questionnaire was English. The research assistant was fully trained for local equivalents of English terminology. Local terms were used where required in the questionnaire for better understanding of the respondents (Additional file 1).

### Sections

The questionnaire was divided into three sections. Section 1 comprised sociodemographic information. Education status was categorized into different groups ranging from uneducated (lowest level) to the masters level and beyond (highest level). Therefore, matriculation, graduation, and masters represented 10, 14, and 16 years of education, respectively. The geographical origin was also determined to ascertain the variety of respondents. Section 2 comprised knowledge regarding the methods used for spiritual therapy. The family member who persuaded the practice of spiritual method was assessed.

Section 3 comprised the attitude towards the practice of spiritual therapy. This was assessed with questions related to caregivers’ beliefs about the effect of spiritual methods on healing, an early cure, and well-being of the children. We also evaluated caregivers’ beliefs about the efficacy of the spiritual methods in curing the disease and if it was possible to become cured with only spiritual methods.

The *Cloths* was defined as a piece of cloth that is used as a wristband, armband, or necklace for safety and *Dam* as an elder/pious person who recites verses from the holy book and blows on the child to spare him from evil while *Nazar wattoo* is a piece of black cloth that is worn as a wristband to circumvent evil. The *Taveez* is the written verses that are encased in leather/metal and worn as an armband or necklace and *Threads* mean that verses are read over threads and the threads are tied around the arms or neck of children to keep them safe from evil.

### Statistical analysis

Data were entered and validated using EpiData version 3.1. A total of 10 % of entries were “double entered” to test data entry quality. We found an error rate of 0.01 %, which was considered as acceptable. The data were cleaned for invalid or out of range values, missing values, and duplicate ID numbers. The data were then imported and analyzed in Windows Statistical Package for Social Sciences (SPSS) version 16.0 using DBMS. Frequencies (percentages) were computed for qualitative variables (e.g., educational status, CAM use, and preferences).

### Ethical considerations

All efforts were made in this study to fulfill the ethical considerations in accordance with the Declaration of Helsinki. The confidentiality of each participant was strictly ensured throughout the survey. The study was presented to the Institutional Review Board of the Institute of Mother and Child Care, Multan, and was approved retrospectively.

## Results

Of the1407 parents/caregivers who were interviewed, 1280 forms were found complete for final analysis. Almost all (1267, 99 %) of the patients resided in Punjab and only 13 (1.0 %) came from Baluchistan, Khyber Pakhtoonkhaw, and Sindh. From Punjab, the majority (817, 63.8 %) were from Multan district followed by Muzaffar Garh (130, 10.2 %), Dera Ghazi Khan (102, 8.0 %), Khanewal (83, 6.5 %), Vehari (48, 3.8 %), and Layyah (30, 2.3 %). A minority (70, 5.4 %) of the respondents came from districts spanning from Peshawar to Karachi.

The majority of the respondents were mothers (1053, 82.4 %) and grandmothers (155, 12.1 %), while only 72 (5.5 %) respondents were others. The maximum numbers of patients were children aged 5–15 years (434, 33.9 %), followed by infants (333, 26 %), children aged 1–5 years (264, 20.6 %), and newborns up to 1 month (249, 19.5 %). The numbers of patients from lower, middle and upper socioeconomic strata were 420 (32.8 %), 601 (47.0 %), and 259 (20.2 %), respectively, as defined by the monthly income of rupees <3000, 3000 to 10,000, and >10,000, respectively (Table 1).

**Table 1:** Association among different characteristics and use of CAM (with percentages in parentheses)

Characteristics	Categories	Use of CAM		Total	*p*-value*
		No	Yes		
Age of Child	Newborn	149 (59.8)	100 (40.2)	249	0.002
	Infant	103 (30.9)	230 (69.1)	333	0.000
	Less than 5 years	111 (42.0)	153 (58.0)	264	0.010
	5 to 15 years	213 (49.1)	221 (50.9)	434	0.701
Economic Status	Less than 3,000	152 (36.2)	268 (63.8)	420	0.000
	3,000 to 10,000	282 (46.9 )	319 (53.1)	601	0.000
	More than 10,000	142 (54.8)	117 (45.2)	259	0.120
Mother’s education	No education	324 (43.0)	430 (57.0)	754	0.000
	Primary	71 (38.8)	112 (61.2)	183	0.002
	Matriculation	89 (44.9)	109 (55.1)	198	0.155
	Graduation	68 (64.2)	38 (35.8)	106	0.004
	Post-graduation	24 (61.5)	15 (38.5)	39	0.015

*p-values are computed for one-sample proportion tests for equality of proportion of using and not using CAM

The majority of mothers were not educated 754 (58.9 %), while the numbers of those with primary (5 years), matric, graduate, and post-graduate education were 183 (14.3 %), 198 (15.5 %), 106(8.3 %), and 39 (3.0 %), respectively. *Taveez* was the most popular CAM irrespective of education level or literacy/illiteracy (65.3–68.4 %) (Table 1).

**Fig. 1:**
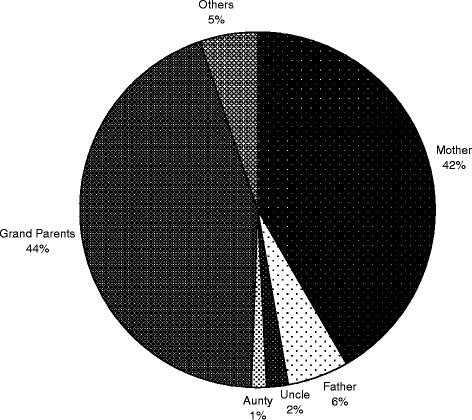
Distribution of the persons, proposing CAM

Grandmothers (312, 44.3 %) and mothers (293, 41.6 %) advised spiritual methods in 605 (85.9 %) children. The father, uncle, aunt, and other relatives were least involved in advising these methods (39, 5.6 %; 15, 2.1 %; 8, 1.1 %; and 37, 5.3 %, respectively (Fig. 1).

A total of 704 (55 %) parents used various spiritual methods. Of these, the numbers of parents who used *Taveez*, *Dam*, *Threads*, *Nazar wattoo*, and *Cloths* were 466 (66.2 %), 40 (5.7 %), 25 (3.6 %), eight (1.1 %), and six (0.9 %), respectively. A total of 159 (22.6 %) parents used more than one method.

Only 100 (40.2 %) parents used CAM for 249 neonates, while almost 70 % (230) of parents used CAM for 333 infants(<1 year). In children aged 1–5 years, more parents used CAM than those who did not, but the percentage of use was lower than that in the infantile period (58 % vs 42 %). Beyond 5 years, the number of parents who used CAM versus those who did not use CAM was almost equal (50.9 % vs 49.1 %, Table 1).

Economic status had a strong bearing on using CAM. With increasing economic status, the use of CAM decreased. Among 420 parents in the lower economic strata, 268 (63.8 %) used CAM, whereas only 117 (45.2 %) of 259 parents from the upper class used CAM. Middle class parents were intermediate, with 53.1 % who used CAM (319/601, Table 1).

There was a significant (p < 0.001, Chi-square test) association between the mother’s education and use of CAM. For illiterate and primary educated mothers, the use of CAM was high (57 % and 61.2 %, respectively), while there was less chance for graduate and post-graduate mothers to use CAM (35.8 % and 38.5 %, respectively, Table 1).

A total of 1269 (99.1 %) respondents believed that allopathic drugs were needed for healing children, alone (776, 60.6 %) or in combination with CAM (493, 38.5 %). Only a small number (11, 0.9 %), mostly mothers (8/11), believed that alternative methods alone would heal the child, and that there was no need for allopathic drugs in curing or treating the child.

A total of 809 (63.2 %) respondents believed that curing the child was possible without use of CAM, while 471 (36.8 %) believed that without the practice of the CAM, a cure would either not be possible at all (277, 21.6 %) or it would be delayed (194, 15.2 %). A total of 276 (34.1 %) of 809 respondents were still using CAM, although they understood that a cure was possible without it (Table 2). However, 43 (9.1 %) of 471 respondents were not using CAM, but they considered that a cure would either not be possible or would be delayed.

**Table 2:** Association between use of CAM and belief about curing

Use of CAM	What happens to curing if you don't use alternate methods?			Total	*p*-value (Chi-square test)
	Possible	Not possible	Delayed		
No	533	19	24	576	0.000
	(92.5 %)	(3.3 %)	(4.2 %)		
Yes	276	258	170	704	
	(39.2 %)	(36.6 %)	(24.1 %)		
Total	809	277	194	1280	

With regard to the question “how much does CAM play in the role of curing the child”, 679 (53.0 %) respondents thought that CAM did not have any part in curing the child. Those who had faith in CAM (601, 47 %) believed that it played a 10 %, 25 %, 50 %, 75 %, or 100 % role in curing the child in 159 (22.5 %), 220 (31.5 %), 195 (23.4 %), 23 (3.2 %), and four (0.5 %) respondents, respectively. Therefore, among those (601) who believed in CAM, 574 (95.5 %) considered that CAM did not play a role of more than 50 % in curing the child.

## Discussion

Parents often ask pediatricians/health care professionals about CAM therapies. However, many pediatricians/health care professionals feel uncomfortable directing patients to these therapies and want further knowledge about CAM therapies [2–4, 18]. The American Academy of Pediatrics provisional section of complementary, holistic, and integrative medicine, a task force on complementary and alternative medicine, stated that “pediatricians and other clinicians who care for children have the responsibility to advise and counsel patients and families about relevant, safe, effective, and age appropriate health services and therapies regardless of whether they are considered mainstream or CAM” [8]. The use of CAM is probably much more common in the population in our study than in many other countries. Therefore, understanding the dynamics of the beliefs of our population regarding CAM is important. Although there are quite a few studies on CAM therapies from Pakistan [10–13, 15, 17, 19], there are no studies regarding the use of spiritual methods by parents for their children. Therefore, the current study results fill this gap in knowledge.

The percentage of people using CAM for their children in the Pakistani community (70.4 % [20], 63.2 % [10], and 55 % in our study) is higher than the reported CAM use in various studies from Western countries (UK, 34 % [21]; The Netherlands, 30 % [22]; United States, ‬12 % [9]). This finding is understandable because our community is predominantly Muslims who have a strong belief in the healing powers of Allah (the ultimate creator of the universe) and the Quran (the book that is the word of Allah and was revealed to the last holy prophet Muhammad). Muslims believe that the ultimate healer is Allah and that the use of drugs is a part of the commandment from Allah for treatment. Therefore, they are likely to use spiritual methods to contribute to healing of the child. Other Eastern countries also share a more spiritual approach, as reported in Sri Lanka (use of CAM is 67.4 %) [23].

In our study, the majority of respondents were mothers, followed by grandmothers. This is because only one woman is allowed to stay with the child in public sector hospitals, while fathers or other men are not allowed to stay. In our public sector hospital/facility, the lower and middle classes comprised the major share of the respondents because these two groups are the main beneficiaries of these facilities.

The majority of the respondents were uneducated or had little education, which is in line with the population’s general literacy level. The popularity of *Taveez* in mothers as a CAM method (66.2 %) in our study is similar to previously reported data from Pakistan (70.4 %) [20], but the majority of the mothers who were post-graduate used *Taveez* (80 %) compared with other methods. Education was a significant contributor towards the attitude of using CAM. Better-educated mothers appear less inclined towards using spiritual methods. This means that education provides enlightenment to these women, whereby they start believing in visible rather than invisible powers of nature and religion. A similar relation with education for CAM use has been previously reported [20].

Mothers appeared to be the most concerned for their children in their infancy as shown by the frequency of use of CAM in this age group (70 %), while its use was less in other age groups. The inverse relationship of CAM use with socioeconomic status has also been previously reported [20]. The main users of spiritual methods were mothers and grandmothers. This is because grandmothers in Pakistani society enjoy a dominant role in terms of respect and obedience. This is why their decisions about treatment modalities for children are respected and obeyed.

There are a few limitations of our study. First, our study described the population of the hospital and its surrounding population. A multicenter study with a broader view of the attitudes of the various communities of the country is required. Second, we targeted the parents of children in the hospital. Further studies need to determine their attitude concerning CAM use in the community. Third, we focused on the maternal side, whereas investigating the paternal attitude may also be interesting. However, this study provides a basis for large scale, multicenter studies. For multiple reasons, including the predominant religious background of our population, spirituality plays a large role in the formation of attitude towards healing. The belief of people in Allah and His powers is pivotal in their use of spiritual methods.

## Conclusions

People using spiritual methods still believe in the use of allopathic medicines. Almost all of them believe that CAM methods play a complementary role with medicines, and healing is least likely without allopathic drugs. Therefore, as long as people use mainstream medicines without these spiritual methods interfering with their health-seeking behaviors, we should not only allow them to do so, we should also encourage them if it increases their level of satisfaction and well-being [3].

## Additional file

Additional file 1:
**Questions & Performa.** (DOCX 16 kb)
