# Supplementary material for: Parental beliefs and practice of spiritual methods for their sick children at a tertiary care hospital of Pakistan- a cross sectional questionnaire study
Source: BMC Complement Altern Med. 2016 Jan 13;16:14. doi: 10.1186/s12906-016-0986-3 (PMC4710972; doi:10.1186/s12906-016-0986-3)
Supplement: Additional file 1: — Questions & Performa. (DOCX 16 kb) [file 12906_2016_986_MOESM1_ESM.docx]

**Questions and Performa:** Respondent: M / D / N / K / P / O

| Sr. No | Reg. No | Date |
| --- | --- | --- |

1. Name & Father’s Name:
2. Dist. Province: P / S / B / N

| 1. **Age of child** | Newborn | Infant | <5 years | 5 – 15 years |
| --- | --- | --- | --- | --- |

| 1. **Socioeconomic** | < 3000 / mo; | < 10,000 / mo | > 10,000 / mo |
| --- | --- | --- | --- |

| 1. **Mother’s education** | Nil | Primary | Matric | Graduate | Masters | Others |
| --- | --- | --- | --- | --- | --- | --- |

1. Alternative Methods:

|  | Taweez | Threads | Clothes | Dum/ Parhai | Nazar watoo |
| --- | --- | --- | --- | --- | --- |
| # |  |  |  |  |  |
| During illness |  |  |  |  |  |

1. Who has proposed these methods?

| Mother | Father | Uncle | Aunty | Grand Parent |  |
| --- | --- | --- | --- | --- | --- |

1. Who will heal the child?

| Drug | Alt. Method | Both |
| --- | --- | --- |

1. Will the child be cured if you don’t use drugs?

| Yes | No |
| --- | --- |

1. What will happen if you don’t use these methods?

| Cure | Possible | Not possible | Delayed | Other |
| --- | --- | --- | --- | --- |

1. How much is the role of alternative methods in healing?

| Nil | 10 % | 25 % | 50 % | 75 % | 100 % |
| --- | --- | --- | --- | --- | --- |

Respondent: M = Mother, D = Dadi, N = Nani, K = Khala, P = Phopho

Province: P = Punjab, S = Sindh, B = Balochistan, N = NWFP
